# Linkage to intensive adherence counselling among HIV-positive persons on ART with detectable viral load in Gomba district, rural Uganda

**DOI:** 10.1186/s12981-021-00349-9

**Published:** 2021-04-20

**Authors:** Rita Nakalega, Nelson Mukiza, Henry Debem, George Kiwanuka, Ronald Makanga Kakumba, Robert Menge, Irene-Kinera Kagimu, Catherine Nakaye, Juliet Allen Babirye, Hellen Kaganzi, Zubair Lukyamuzi, Samuel Kizito, Cynthia Ndikuno Kuteesa, Andrew Mujugira

**Affiliations:** 1 Makerere University-Johns Hopkins University (MU-JHU) Research Collaboration Kampala, Kampala, Uganda; 2 School of Public Health, College of Health Sciences, Makerere University, Kampala, Uganda; 3 Department of Public Health and Preventive Medicine, School of Medicine, University of Liverpool, Liverpool, UK; 4 MRC/UVRI & LSHTM Uganda Research Unit, Kampala, Uganda; 5 School of Social Sciences, College of Humanities and Social Sciences, Makerere University Kampala, Kampala, Uganda; 6 Department of Global Health, School of Public Health, Boston University, Boston, USA; 7 Department of Epidemiology and Biostatistics, School of Medicine, Makerere University College of Health Sciences, Kampala, Uganda; 8 Infectious Diseases Institute, College of Health Sciences, Makerere University, Kampala, Uganda

**Keywords:** Intensive adherence counselling, Viral load, ART, HIV, Uganda

## Abstract

**Background:**

Antiretroviral therapy (ART) adherence is a primary determinant of sustained viral suppression, HIV transmission risk, disease progression and death. The World Health Organization recommends that adherence support interventions be provided to people on ART, but implementation is suboptimal. We evaluated linkage to intensive adherence counselling (IAC) for persons on ART with detectable viral load (VL).

**Methods:**

Between January and December 2017, we conducted a retrospective chart review of HIV-positive persons on ART with detectable VL (> 1000 copies/ml), in Gomba district, rural Uganda. We abstracted records from eight HIV clinics; seven health center III’s (facilities which provide basic preventive and curative care and are headed by clinical officers) and a health center IV (mini-hospital headed by a medical doctor). Linkage to IAC was defined as provision of IAC to ART clients with detectable VL within three months of receipt of results at the health facility. Descriptive statistics and multivariable logistic regression analyses were used to evaluate factors associated with linkage to IAC.

**Results:**

Of 4,100 HIV-positive persons on ART for at least 6 months, 411 (10%) had detectable VL. The median age was 32 years (interquartile range [IQR] 13–43) and 52% were female. The median duration on ART was 3.2 years (IQR 1.8–4.8). A total of 311 ART clients (81%) were linked to IAC. Receipt of ART at a Health Center level IV was associated with a two-fold higher odds of IAC linkage compared with Health Center level III (adjusted odds ratio [aOR] 1.78; 95% CI 1.00–3.16; p = 0.01). Age, gender, marital status and ART duration were not related to IAC linkage.

**Conclusions:**

Linkage to IAC was high among persons with detectable VL in rural Uganda***, ***with greater odds of linkage at a higher-level health facility. Strategies to optimize IAC linkage at lower-level health facilities for persons with suboptimal ART adherence are needed***.***

## Introduction

Antiretroviral therapy (ART) adherence is a modifiable determinant of viral suppression and long-term treatment success i.e., viral load (VL) suppression below the lower limit of detection of commercially available assays [1–4]. Persons with suboptimal ART adherence have sub-therapeutic drug levels and are three times as likely to rapidly progress to virologic failure compared with those who adhere to ART [5, 6]. Barriers to ART adherence include number and timing of doses, pill size, side-effects, transportation costs to the HIV clinic, depression and substance abuse [7]. Targeted adherence interventions, including adherence counselling, improve viral suppression in 57–84% of individuals with detectable viraemia [8–10]. Failure to achieve viral suppression after adherence counseling is assumed to indicate virologic failure in settings where drug resistance testing is unavailable [11]. Studies suggest that approximately 70% of persons on first-line ART who have a first high viral load will re-suppress following an adherence intervention [8], suggesting that non-adherence is a key reason for unsuppressed viral load.

The World Health Organization (WHO) recommends that HIV-positive persons with detectable VL (> 1000 copies/ml), be offered enhanced adherence counselling [12]. In 2016, the government of Uganda implemented intensive adherence counseling (IAC) for persons with detectable HIV viral load in accordance with WHO recommendations [13]. The goal of IAC is to help clients identify and gain insight into their specific adherence barriers, explore strategies to overcome these barriers and to formulate a comprehensive adherence plan to help improve ART adherence [12, 14]. An IAC multidisciplinary team comprises of counsellors, clinicians, family members, nurses, peers, nutritionists and psychiatrists who address multi-factorial issues that influence adherence including nutrition, stigma and disclosure [12, 13]. The IAC package includes the provision of individual needs assessment, education sessions and adherence counselling to patients and the use of 5As—Assess, Advise, Agree, Assist and Arrange–when offering adherence support to people with detectable viraemia [13].

For all those with detectable VL, Uganda guidelines recommend 3 IAC sessions, 1 month apart, with repeat viral load testing one month after the 3rd IAC session [15]. Those with persistent virologic failure after IAC are considered for switching to second line treatment provided that all adherence challenges have been addressed [15]. Since its launch in Uganda in 2016, implementation of IAC has been suboptimal. An analysis of 449 children and adolescents with detectable VL (> 1,000 copies/ml) found that 77% completed all 3 IAC sessions, 16% received one or two sessions and 7% were not linked to IAC within 400 days of receiving a viral load result [16]. Little is known about linkage to IAC for adults in sub-Saharan Africa and in rural settings. Suboptimal adherence is a major challenge globally because of a diversity of person- and facility-level factors. Characterizing factors affecting IAC implementation will help improve HIV service delivery. The objective of this study was to evaluate IAC linkage among HIV-positive persons on ART with detectable viral loads in Gomba District, Uganda.

## Materials and methods

### Subjects and setting

We conducted a retrospective cohort study of HIV-positive persons who received ART between January and December 2017 at 8 HIV clinics in Gomba district, rural Uganda [17]. Gomba, one of 136 administrative districts in Uganda, has a population of 160,075 of which 92% is rural and 49% are female [18]. Uganda’s healthcare system operates on a referral system, in which lower-level facilities refer cases to the next level unit. Primary care facilities are organized by administrative division and include Health Center II (parish), Health Center III (sub-county) and Health Center IV (county). Gomba district had one Health Center IV (a mini hospital with surgical and obstetric services managed by a medical doctor) and 7 Health Center III facilities (each with a general outpatient clinic and maternity ward and led by clinical officers) [19]. In 2016, Uganda adopted WHO recommendations to offer immediate ART to all persons newly diagnosed with HIV [13]. Following ART initiation, VL testing is performed after 6 months, and if virally suppressed, 12 months thereafter [13]. At scheduled clinic visits, socio-demographic and clinical data are recorded on client treatment cards (blue card) and clients are asked to return to clinic every three months for adherence assessment and ART refills. At the scheduled visit for VL testing, the date of blood sample collection is documented on the client’s blue card. Samples from the 8 ART clinics are warehoused at a district laboratory hub prior to transportation to the national reference laboratory. Facility staff document VL results in viral load registers and on client blue cards at subsequent clinic visits [17].

### Population and procedures

Clients with detectable VL received IAC every month and a minimum of three IAC sessions were required prior to repeat viral load testing which was performed at the third IAC visit [17]. At the health center IV, counselling was provided by health care workers including adherence counsellors, nurses and clinical officers. At the health center III, IAC provision was supported by peers (expert clients with HIV or members of village health teams) due to limitations in the numbers of health workers. Peers were trained and supervised by health workers during provision of IAC. Data were collected from all 8 ART clinics during a 6-week period from mid-October to November 2018. We included all clients with detectable VL at all eight health facilities. Each client was given a unique identifier and data abstracted from viral load registers, IAC registers and client treatment cards that contain a record of client details. These documents were completed at each clinic visit by the health facility staff. Data on age, sex, marital status, duration on ART, and dates of viral load testing, receipt of test results and first IAC session, the number of IAC sessions and health facility level were entered into a Microsoft® Excel database. Quality control checks were performed to ensure data quality.

### Laboratory methods

Viral load testing was performed in batch at the Central Public Health Laboratory as standard of care using the COBAS® AmpliPrEP/COBAS® TaqMan® HIV-1 Test, v2.0 kit (Roche Molecular System, Inc, Pleasanton, CA, USA). Viral load test results were returned to ART clinics within 6–8 weeks.

### Statistical analysis

Linkage to IAC (the primary outcome) was defined as provision of IAC to an HIV-positive person with detectable VL within three months of receipt of VL viral load results at the health facility. Detectable VL was defined as HIV RNA concentration > 1,000 copies/ml [11]. Time to first IAC session was was counted from the date of receipt of VL test results at the health facility to date of first IAC session. We used descriptive analytical methods and a multivariable logistic regression model to assess factors related to lAC linkage. Pearson’s chi-square test was used to test relationships between categorical variables. Factors with p ≤ 0.2 in bivariate analyses were included in the multivariable model. P-values ≤ 0.05 were considered statistically significant. Data were analyzed using Statistical Package for the Social Sciences (SPSS) version 20.0 (IBM, Corp. Armonk, NY, USA).

### Ethics statement

The study was approved by the University of Liverpool Board of Ethics (*H00057734*), Mildmay Uganda Research Ethics Committee (*0208–2018*) and Uganda National Council for Science and Technology (*HS255ES*). Administrative clearance was obtained from the District Health Officer of Gomba district prior to study implementation.

## Results

### Participant characteristics

A total of 4,100 clients had received ART for at least six months during the study period. Of these, 411 (10%) had detectable VL (> 1000 copies/mL) and were included in the retrospective analysis. The median age was 32 years (interquartile range [IQR] 13–43) and 52% were female (Table 1). The median duration on ART was 3.2 years (IQR 1.8–4.8), and most (63%) received ART services at a Health Center level III facility.

**Table 1:** Participant characteristics and correlates of IAC linkage

Characteristic	N (%)	Linked to IAC N (%)	Bivariate analysis		Multivariable analysis	
			OR (95% CI)	p-value	AOR (95% CI)	p-value
Age group						
1–15 years	115 (28.0)	90 (78.3)	Referent			
16–30 years	78 (28.0)	62 (79.5)	0.84 (0.53, 2.18)	0.84		
31–45 years	133 (32.4)	108 (81.2)	0.57 (0.65, 2.23)	0.57		
46 + years	85 (20.7)	71 (83.5)	0.35 (0.68, 2.91)	0.35		
Duration of ART						
6–11.9 months	42 (10.2)	32 (76.2)	Referent		Referent	
1–5 years	287 (69.8)	226 (78.7)	1.16 (0.54, 2.49)	0.71	1.12 (0.64, 1.94)	0.70
> 5 years	82 (20.0)	73 (89.0)	2.54 (0.94, 6.83)	0.07	1.64 (0.68, 3.94)	0.28
Health facility level						
Health IV	154 (37.5)	134 (87)	2.04 (1.18, 3.54)		1.78 (1.00, 3.16)	
Health III	257 (62.5)	197 (76.7)	Referent	0.01	Referent	0.01
Gender						
Male	197 (47.9)	169(79.0)	Referent			
Female	214 (52.1)	162 (82.2)	1.23 (0.75, 2.02)	0.41		
Marital status						
Married	41 (10.0)	33 (80.5)	Referent			
Divorced/Separated	163 (39.7)	57 (81.4)	1.05 (0.44, 2.48)	0.92		
Single	70 (17.0)	130 (79.8)	1.11 (0.55, 2.27)	0.77		
Widowed/Widower	22 (5.4)	21 (95.5)	5.33 (0.69, 41.88)	0.11		

### Linkage to IAC

Three hundred and thirty one participants (81%) with non-suppressed viraemia were linked to the IAC intervention within three months of receipt of viral load test results. Sixteen percent (65/411) had not been linked to IAC by the time of the study, and three percent (15/411) had been linked to IAC after more than three months. Nearly all widowed participants (96%) were linked to IAC. Linkage was similar among participants who had been on ART for more than 5 years and those on ART for 6–11.9 months (89% vs. 76%; p = 0.07). A higher proportion of participants receiving HIV care at Health Center IV were linked to IAC than Health Center III ART recipients (87% vs 77%; p = 0.01). Similar proportions of women and men (82% and 79%, respectively) were linked to IAC.

In bivariate analysis, clients receiving ART at Health Center IV facilities had two-fold higher odds of linkage to IAC compared with Health Center III (odds ratio [OR] 2.04 (95% confidence interval [CI]: 1.18–3.54; p = 0.01) (Table 1). There was a non-statistically significant trend for IAC linkage among those with ART duration > 5 years (odds ratio [OR] 2.54; 95% CI 0.94–6.83; p = 0.07). In multivariable analyses, receipt of ART at a Health Center level IV was significantly associated with linkage to IAC (adjusted OR 1.78; 95% CI 1.00–3.16; p = 0.01). Age, gender, marital status and ART duration were not related to IAC linkage.

## Discussion

In this retrospective cohort study of 4,100 HIV-positive women and men in rural Uganda, one-in-ten had detectable viral load. Eighty one percent were linked to the IAC intervention within 3 months of receipt of viral load results at the HIV care facility. Receipt of HIV treatment at a higher level public health facility was the only factor associated with linkage to IAC. Linkage was similar with respect to age, gender, marital status and duration of ART.

We found that persons receiving HIV care at a Health Center IV had two-fold higher odds of linkage to IAC compared to Health Center III, perhaps because Health Center IV staff conducted IAC themselves whereas patients at Health Center III facilities were referred to peer counsellors [17]. This extra step in linkage to IAC may have increased the odds of non-linkage. Additionally, peer counsellors were sometimes unavailable due to other health system support roles they performed in the community. Improving linkage to IAC at lower-level health facilities is needed to improve HIV treatment outcomes in Uganda. Prompt linkage to IAC and routine VL monitoring to confirm treatment response facilitates faster regimen switch to second line ART in a setting where genotypic testing to inform choice of efficacious drugs is not standard of care because of high cost [20–22]. We found that gender was not associated with linkage to IAC. Similar findings have been reported from South Africa where gender was not associated with uptake of HIV treatment [23].

IAC is also known as enhanced adherence counselling (EAC) elsewhere in sub-Saharan Africa where it is standard of care for patients failing first- or second-line ART. A study in Uganda found that IAC was ineffective, with 91% of persons on first-line ART remaining virologically unsuppressed (VL > 1000 copies/ml) after 3 months [24]. In contrast, a study in South Africa found that enhanced adherence counselling was effective for patients unsuppressed on second-line ART [25]. In this study, in which 64% were resuppressed after 3 months, EAC consisted of at least one counseling session with an experienced adherence counsellor or social worker and follow up visits with a medical officer experienced in treatment failure until they re-suppressed. Similar findings were observed in Burkina Faso, Ivory Coast, Mali and Senegal, in West Africa, where 67% of patients re-suppressed on second line ART after 4 months [26] In this study, IAC consisted of monthly counselling sessions coupled with any of the following strategies: directly observed therapy by a pill buddy, a pill organizer, weekly phone calls, daily alarm reminders, daily text messages, home visits, or peer self-help groups. IAC curricula and implementation strategies vary among countries and health systems in sub-Saharan Africa and may account for these divergent results [27].

The proportion linked to IAC in our study is similar to other work in sub-Saharan Africa. In an evaluation of 489 people with plasma viral loads  > 1,000 copies/ml in Zimbabwe, 83% attended all three enhanced adherence counselling sessions, and 47% re-suppressed [28]. Although we did not evaluate viral suppression as a measure of the effectiveness of IAC, attendance of all 3 adherence sessions in the Zimbabwean study was independently associated with higher probability of viral suppression. In the ADVANCE trial in South Africa, in which viral suppression with dolutegravir plus either tenofovir alafenamide or tenofovir disproxil fumarate was non-inferior to standard of care, adherence counseling led to resuppression of viraemia [29]. Two studies in South Africa found that following IAC, viral re-suppression occurred in 68% and 61% of persons on ART, respectively [25, 30]. A study in rural Uganda found that 60% of HIV-positive persons with detectable viraemia achieved viral suppression after adherence counselling interventions [31]. These findings suggest that most treatment failure in this setting is associated with non-adherence and can be addressed with targeted adherence support [32].

The strengths of our study include the district-wide assessment of linkage to IAC in a rural setting, and the large sample size with similar proportions of women and men which improves the generalizability of our findings. Our study has limitations. We did not conduct viral load or drug resistance testing after IAC and are unable to assess the effectiveness of IAC in this setting. However, drug resistance testing is not routinely available. Finally, we did not evaluate counseling quality or fidelity to IAC counseling materials in this programmatic setting.

In conclusion, linkage to IAC was high among persons with detectable viral load in rural Uganda. Receiving services from Health Center level IV was associated with increased odds of linkage to IAC.Strategies to optimize IAC linkage at lower-level health facilities for persons with suboptimal ART adherence are needed. Future studies should or conduct qualitative research to explore participant and provider perceptions and experiences with IAC and generate actionable data for program improvement.

## Data Availability

The dataset used and analyzed during this study is available from the corresponding author upon request.

## Abbreviations

IAC: Intensified adherence counselling
ART: Anti-retroviral treatment
HIV: Human immunodeficiency virus
VL: Viral load
WHO: World Health Organization
